# Maxillary Arch Width and Buccal Corridor Changes Following Extraction and Non-Extraction Fixed Orthodontic Treatment: A Comparative Study

**DOI:** 10.7759/cureus.113699

**Published:** 2026-07-30

**Authors:** Ardhra C A, Virendra Vadher, Shweta Singh, Arvind Nair, Gangesh Bahadur Singh, Pratibha Saini

**Affiliations:** 1 Department of Orthodontics and Dentofacial Orthopaedics, Government Dental College and Hospital, Raipur, IND

**Keywords:** buccal corridor, dental arch, extraction treatment, fixed orthodontic appliances, maxillary arch width, non-extraction treatment, orthodontics, premolar extraction, smile esthetics, transverse arch dimensions

## Abstract

Introduction

The effects of premolar extraction on maxillary arch width and buccal corridor size are a debatable topic in orthodontics. It has been suggested that extraction treatment can lead to reduction in dental arch width and more buccal corridor presentation, which can impact smile aesthetics. The aim of this study was to compare the differences in maxillary arch width and buccal corridor dimensions after extraction and non-extraction fixed orthodontic therapy.

Materials and methods

It was a retrospective study involving pre-treatment and post-treatment records of patients treated with contemporary fixed orthodontic appliances in the Department of Orthodontics and Dentofacial Orthopaedics, Government Dental College, Raipur. A total of 70 patients (35 extraction and 35 non-extraction) were screened initially. After a photographic comparability criterion in terms of intercanthal-to-inter-commissural width ratio was applied, 48 subjects were used in the final analysis (25 extraction and 23 non-extraction). Maxillary inter-canine, inter-premolar, and intermolar widths were measured on the study models using a digital caliper. Buccal corridor dimensions were evaluated from frontal smile photographs using Adobe Photoshop software. Paired Student's *t*-test was used for intragroup comparisons, and independent Student's *t*-test was used to compare treatment-induced changes between the extraction and non-extraction groups.

Results

Significant increases in intercanine and interpremolar widths were observed following treatment in both groups (p<0.05). A significant increase in intermolar width was observed only in the non-extraction group (p=0.008). Intergroup comparison demonstrated a significantly greater intermolar width change in the non-extraction group (p=0.001), whereas intercanine and interpremolar width changes were comparable between the groups. Buccal corridor measurements remained largely unchanged following treatment, with no significant intergroup differences. A statistically significant increase in the IL:SW (interlast visible maxillary teeth distance (IL) and smile width (SW)) ratio was observed in the non-extraction group (p=0.025).

Conclusions

Both extraction and non-extraction fixed orthodontic treatment were associated with favourable transverse arch changes, while buccal corridor dimensions remained largely stable following treatment. Intergroup comparison demonstrated a significant difference only in intermolar width changes, whereas the remaining transverse and buccal corridor measurements were comparable between the groups. These findings suggest that premolar extraction therapy does not necessarily result in clinically unfavourable narrowing of the maxillary arch or increased buccal corridor dimensions when contemporary fixed orthodontic treatment mechanics are employed.

## Introduction

Smile aesthetics has become an integral component of contemporary orthodontic treatment. While traditional orthodontic therapy primarily focused on achieving optimal occlusion and functional efficiency, increasing emphasis is now placed on facial appearance and smile harmony. As patients become more conscious of facial aesthetics, the influence of orthodontic treatment on smile characteristics has gained considerable clinical importance [1,2].

Among the various components of smile aesthetics, the buccal corridor has received significant attention. The buccal corridor refers to the negative space visible between the buccal surfaces of the posterior teeth and the corners of the mouth during smiling. It contributes to the perception of smile fullness and is considered an important parameter in smile characteristics [3-5].

The issues surrounding the correlation between the buccal corridors and the attractiveness of the smile are debatable. Some researchers have suggested that the wider the smile and the narrower the buccal corridors, the more attractive the smile is viewed to be, whilst others have found no significant impact of buccal corridor size on total smile aesthetics [4,6,7]. Despite these differing opinions, buccal corridor evaluation continues to be an important aspect of orthodontic diagnosis and treatment assessment.

One of the most widely used treatment methods in orthodontics is premolar extraction, which is considered an effective treatment method for correcting crowding, dentoalveolar protrusion and tooth size-arch length discrepancies [8]. Nevertheless, the possible impact of extraction therapy on the smile features has been questioned. It has been proposed that the extraction of premolar teeth can cause a decrease in maxillary dental arch width, which in turn causes a buccal corridor to be wider and results in a less aesthetic smile [9]. As a result, the effects of extraction and non-extraction treatment on the transverse arch size and the characteristics of the buccal corridors have been a subject of much controversy.

An important parameter in orthodontic diagnosis, treatment planning and post-treatment stability is maxillary arch width. A number of studies have compared changes in arch width after extraction and non-extraction treatment, but the results were not convergent [3,9-11]. These variations could be due to differences in treatment mechanics, sample characteristics and measurement techniques. Furthermore, previous investigations have used different methods to assess buccal corridors, including linear measurements, proportional ratios and photographic analyses, making direct comparisons between studies difficult [3,12].

Recent evidence continues to highlight the ongoing controversy regarding the effects of extraction and non-extraction orthodontic treatment on dental arch dimensions and smile characteristics [3,13,14]. Although numerous studies have investigated either arch width changes or buccal corridor dimensions, limited evidence is available evaluating both parameters simultaneously in patients treated with fixed orthodontic appliances. Therefore, the present study was undertaken to evaluate and compare changes in maxillary arch width and buccal corridor dimensions following extraction and non-extraction fixed orthodontic treatment.

## Materials and methods

Study design and sample selection

This retrospective comparative study was conducted in the Department of Orthodontics and Dentofacial Orthopaedics, Government Dental College, Raipur, after obtaining approval from the Institutional Scientific and Ethical Committee (No. ECR/22/GDC/CG/2024).

Pre-treatment and post-treatment orthodontic records, including frontal smile photographs and maxillary study casts, of patients treated with contemporary fixed orthodontic appliances were screened from the departmental archives and from patients who completed orthodontic treatment during the study period. Patients fulfilling the predefined inclusion and exclusion criteria and having complete orthodontic records were selected based on the availability of suitable records. Eligible subjects were allocated to the extraction or non-extraction group according to the treatment received. An initial sample of 70 patients was selected, comprising 35 patients treated with extraction of four premolars and 35 patients treated without extractions.

To ensure comparability between pre-treatment and post-treatment smile photographs, the ratio of intercanthal width to intercommissural width was calculated according to the method described by Meyer et al. [3]. Subjects with a ratio difference of 0.04 or less between pre-treatment and post-treatment photographs were considered photographically comparable and included in the final analysis [3]. Following this screening procedure, the final sample consisted of 48 patients, including 25 patients in the extraction group and 23 patients in the non-extraction group.

Patients with complete permanent dentition and complete pre-treatment and post-treatment orthodontic records were included in the study. In the extraction group, patients had undergone extraction of four premolars, one in each quadrant. Patients with congenitally missing teeth (except third molars), craniofacial anomalies, previous treatment with palatal expansion appliances, or incomplete orthodontic records were excluded.

Sample size calculation

Sample size estimation was performed using G*Power software version 3.1 (Heinrich-Heine-Universität Düsseldorf, Düsseldorf, Germany) based on the findings of Meyer et al. [3]. Assuming an effect size of 0.63, an alpha error probability of 0.05, and a study power of 95%, the minimum required sample size was calculated to be 35 subjects per group.

Photographic evaluation

Pre-treatment and post-treatment posed frontal smile photographs were analysed using Adobe Photoshop CS3 (Adobe Systems Inc., San Jose, CA, USA). Buccal corridor measurements were obtained according to the protocol described by Meyer et al. [3].

Linear measurements included intercanine distance (IC), interlast visible maxillary teeth distance (IL), and smile width (SW). The ratios IC:SW and IL:SW were calculated for each subject.

Area measurements included buccal corridor area relative to the canines (BCC), buccal corridor area relative to the last visible maxillary teeth (BCL), and total smile area (TSA). The ratios BCC:TSA and BCL:TSA were calculated for each subject.

Cast evaluation

Pre-treatment and post-treatment maxillary study casts were evaluated using a digital caliper. The arch widths were measured at the maxillary canine, anterior premolar and first molar regions. The measurements were all in millimeters.

Statistical analysis

The data obtained were entered in Microsoft Excel 2007 (Microsoft Corporation, Redmond, WA, USA) and were analysed in the Statistical Package for the Social Sciences (SPSS) software version 16.0 (SPSS Inc., Chicago, IL, USA).

Descriptive statistics including mean and standard deviation were calculated for all variables. Intragroup comparisons between pre-treatment and post-treatment measurements were performed using the paired Student’s *t*-test. Intergroup comparisons between the extraction and non-extraction groups were performed using the independent Student’s t-test. A *p*-value of less than 0.05 was considered statistically significant.

## Results

Of the initial 70 subjects screened, 48 satisfied the photographic comparability criterion and were included in the final analysis. The final sample comprised 25 subjects treated with premolar extraction (Group 1) and 23 subjects treated without extraction (Group 2) (Table 1).

**Table 1:** Distribution of subjects included in the final analysis. n=number of subjects. Percentages are based on the final study sample (N=48).

Group	n (%)
Extraction (Group 1)	25 (52.1)
Non-extraction (Group 2)	23 (47.9)
Total	48 (100)

In the extraction group, statistically significant increases were observed in intercanine and interpremolar widths following treatment, whereas intermolar width did not change significantly (Table 2).

**Table 2:** Pretreatment (T1) and posttreatment (T2) maxillary arch width measurements in the extraction group (n=25). T1=pre-treatment; T2=post-treatment; SD=standard deviation. Paired Student's *t*-test was used for intragroup comparisons.

Variable	T1 Mean±SD	T2 Mean±SD	*p *value
Intercanine width	33.4±3.14	34.5±2.67	0.006
Inter-premolar width	39.5±3.04	43.4±2.55	< .001
Inter-first molar width	49.8±2.65	49.2±2.78	0.07

In the non-extraction group, statistically significant increases were observed in intercanine, interpremolar, and intermolar widths following treatment (Table 3).

**Table 3:** Pretreatment (T1) and posttreatment (T2) maxillary arch width measurements in the non-extraction group (n=23). T1=pretreatment; T2=posttreatment; SD=standard deviation. Paired Student's *t*-test was used for intragroup comparisons.

Variable	T1 Mean±SD	T2 Mean±SD	*p* value
Intercanine width	34±3.22	35.6±1.76	0.01
Interpremolar width	41.2±2.97	43.9±1.88	< .001
Inter-first molar width	51.3±2.76	52.4±2.23	0.008

The evaluation of buccal corridor measurements revealed no significant changes in the IC:SW, BCC:TSA or BCL:TSA ratios in either group. A statistically significant increase in the IL:SW ratio was observed only in the non-extraction group following treatment (p=0.025) (Table 4).

**Table 4:** Pretreatment (T1) and posttreatment (T2) buccal corridor measurements in the extraction (n = 25) and non-extraction (n = 23) groups. T1=pretreatment; T2=posttreatment; SD=standard deviation; IC=intercanine distance; IL=interlast visible maxillary teeth distance; SW=smile width; BCC=buccal corridor area relative to the canines; BCL=buccal corridor area relative to the last visible maxillary teeth; TSA=total smile area. Paired Student's *t*-test was used for intragroup comparisons.

Parameter	Group	T1 Mean±SD	T2 Mean±SD	*p* value
IC:SW	Extraction	0.68±0.04	0.67±0.04	0.305
IC:SW	Non-extraction	0.67±0.06	0.66±0.14	0.902
IL:SW	Extraction	0.80±0.06	0.81±0.04	0.839
IL:SW	Non-extraction	0.79±0.04	0.84±0.09	0.025
BCC:TSA	Extraction	0.17±0.06	0.17±0.07	1
BCC:TSA	Non-extraction	0.23±0.09	0.31±0.21	0.086
BCL:TSA	Extraction	0.08±0.04	0.08±0.03	0.767
BCL:TSA	Non-extraction	0.09±0.05	0.09±0.09	0.861

Intergroup comparison of treatment-induced changes in maxillary arch width demonstrated no statistically significant differences in intercanine (p=0.530) or interpremolar width changes (p=0.119) between the extraction and non-extraction groups. However, the change in intermolar width differed significantly between the groups (p=0.001) (Table 5).

**Table 5:** Intergroup comparison of treatment-induced changes in maxillary arch width between the extraction (n = 25) and non-extraction (n = 23) groups. Independent Student's *t*-test was used for intergroup comparisons.

Variable	Extraction (Mean difference ± SD)	Non-extraction (Mean difference ± SD)	*p*-value
Intercanine width	-1.16±1.94	-1.59±2.71	0.530
Interpremolar width	-3.87±2.30	-2.78±2.46	0.119
Intermolar width	0.62±1.65	-1.10±1.80	0.001

Intergroup comparison of treatment-induced changes in buccal corridor measurements demonstrated no statistically significant differences between the extraction and non-extraction groups for IC:SW (p=0.738), IL:SW (p=0.102), BCC:TSA (p=0.093) or BCL:TSA (p=0.962) ratios (Table 6).

**Table 6:** Intergroup comparison of treatment-induced changes in buccal corridor measurements between the extraction (n = 25) and non-extraction (n = 23) groups. IC=intercanine distance; IL=interlast visible maxillary teeth distance; SW=smile width; BCC=buccal corridor area relative to the canines; BCL=buccal corridor area relative to the last visible maxillary teeth; TSA=total smile area; SD=standard deviation. Independent Student's *t*-test was used for intergroup comparisons.

Parameter	Extraction (Mean difference±SD)	Non-extraction (Mean difference±SD)	*p*-value
IC:SW	0.015±0.073	0.004±0.150	0.738
IL:SW	-0.004±0.088	-0.048±0.096	0.102
BCC:TSA	0.000±0.098	-0.086±0.229	0.093
BCL:TSA	0.003±0.047	0.004±0.106	0.962

## Discussion

The present study evaluated changes in maxillary arch width and buccal corridor dimensions following extraction and non-extraction fixed orthodontic treatment. Significant increases in intercanine and interpremolar widths were observed following treatment in both groups, whereas a significant increase in intermolar width was observed only in the non-extraction group. Intergroup comparison of treatment-induced changes demonstrated a significant difference only for intermolar width, while changes in intercanine and interpremolar widths were comparable between the groups. Buccal corridor measurements remained largely unchanged following treatment, with the exception of the IL:SW ratio in the non-extraction group. These findings suggest that contemporary fixed orthodontic treatment can produce favourable transverse arch changes while maintaining overall smile characteristics.

The increase in intercanine and interpremolar widths observed in both groups is consistent with previous investigations reporting maintenance or expansion of transverse arch dimensions following orthodontic treatment. Meyer et al. [3], Kim and Gianelly [9], Aksu and Kocadereli [10], Isik et al. [11], Oz et al. [15], and Hegde et al. [16] similarly reported that premolar extraction treatment does not inevitably result in narrowing of the maxillary arch. The observed increase in intermolar width in the non-extraction group may be attributed to treatment mechanics aimed at transverse arch development and alignment. Furthermore, intergroup comparison demonstrated a significantly greater intermolar width change in the non-extraction group, whereas intercanine and interpremolar width changes were comparable between the two groups. These findings suggest that treatment modality may have influenced posterior arch width changes, while transverse changes in the remaining regions were comparable between the extraction and non-extraction groups.

The relationship between extraction therapy and buccal corridor size has been a contentious topic in orthodontics [4,7]. It has been suggested that premolar extraction may result in a narrower maxillary dental arch, leading to wider buccal corridors and reduced smile aesthetics [4,9]. However, the findings of the present study did not support this assumption. Most buccal corridor measurements remained stable following treatment in both groups, and intergroup comparison of treatment-induced changes demonstrated no significant differences between the extraction and non-extraction groups. These findings are consistent with those of Meyer et al. [4], Kim and Gianelly [9], Akyalcin et al. [12], Sarkar et al. [17] and Bühling et al. [18], who likewise reported minimal or no clinically significant adverse effects of extraction therapy on smile fullness or buccal corridor dimensions. Collectively, these findings suggest that buccal corridor appearance is influenced by multiple factors, including arch form, smile width, tooth position and treatment mechanics, rather than extraction status alone [7].

The significant increase in the IL:SW ratio observed in the non-extraction group suggests an increase in the visible maxillary dentition during smiling. However, this isolated finding was not accompanied by significant changes in the remaining buccal corridor parameters or by significant intergroup differences. Although several transverse arch width measurements reached statistical significance, these changes were not accompanied by corresponding changes in buccal corridor dimensions or overall smile aesthetics. Therefore, the observed transverse changes are unlikely to produce clinically perceptible differences in smile appearance or substantially influence clinical decision-making when contemporary fixed orthodontic treatment mechanics are employed. Overall, these findings indicate that statistically significant transverse arch changes did not translate into clinically perceptible differences in smile aesthetics, suggesting that the choice between extraction and non-extraction treatment should be based on comprehensive diagnostic considerations rather than concerns regarding smile fullness alone [7].

From a clinical perspective, the present findings suggest that premolar extraction, when performed using contemporary fixed orthodontic treatment mechanics, does not necessarily compromise transverse maxillary arch dimensions or smile aesthetics. These findings support basing the choice between extraction and non-extraction treatment on individualized diagnostic and biomechanical considerations, without undue concern regarding adverse effects on smile fullness.

The retrospective design, the use of two-dimensional photographic analysis, and the absence of long-term follow-up should be considered when interpreting the findings of the present study. Although the sample size calculation indicated a minimum requirement of 35 subjects per group, application of the photographic comparability criterion reduced the final sample to 25 and 23 subjects, respectively. Therefore, smaller intergroup differences may not have been detected. Because multiple statistical comparisons were performed, the possibility of type I error cannot be completely excluded. Future prospective studies with larger sample sizes and long-term follow-up are recommended to further clarify the relationship between orthodontic treatment, transverse arch dimensions, and smile aesthetics.

## Conclusions

Both extraction and non-extraction fixed orthodontic treatment were associated with significant increases in maxillary arch width following treatment, while buccal corridor dimensions remained largely unchanged in both groups. Intergroup comparison demonstrated a significant difference only in intermolar width changes, whereas the remaining transverse arch and buccal corridor measurements were comparable between the groups. These findings suggest that premolar extraction therapy does not necessarily result in narrowing of the maxillary dental arch or unfavorable changes in buccal corridor dimensions when contemporary fixed orthodontic treatment mechanics are employed. Therefore, extraction decisions should be guided by individual diagnostic and treatment requirements rather than concerns regarding adverse effects on smile aesthetics.
